# Allergic rhinitis, atopic dermatitis, and asthma are associated with differences in school performance among Korean adolescents

**DOI:** 10.1371/journal.pone.0171394

**Published:** 2017-02-16

**Authors:** So Young Kim, Min-Su Kim, Bumjung Park, Jin-Hwan Kim, Hyo Geun Choi

**Affiliations:** 1 Department of Otorhinolaryngology-Head & Neck Surgery, CHA Bundang Medical Center, CHA University, Seongnam, Korea; 2 Department of Otorhinolaryngology-Head & Neck Surgery, Korea University Ansan Hospital, Ansan, Korea; 3 Department of Otorhinolaryngology-Head & Neck Surgery, Hallym University College of Medicine, Anyang, Korea; 4 Department of Otorhinolaryngology-Head & Neck Surgery, Hallym University College of Medicine, Seoul, Korea; Beijing Tongren Hospital, CHINA

## Abstract

Several studies have reported negative relations between allergic diseases and school performance but have not simultaneously considered various allergic diseases, including allergic rhinitis, asthma, and atopic dermatitis, and only examined a limited number of participants. The present study investigated the associations of allergic rhinitis, asthma, and atopic dermatitis with school performance in a large, representative Korean adolescent population. A total of 299,695 7th through 12th grade students participated in the Korea Youth Risk Behaviour Web-based Survey (KYRBWS) from 2009 to 2013. The subjects’ history of allergic rhinitis, asthma, and atopic dermatitis and number of school absences due to these diseases in the previous 12 months were examined and compared. School performance was classified into 5 levels. The relations between allergic disorders and school performance were analyzed using multiple logistic regressions with complex sampling and adjusted for the subjects’ durations of sleep, days of physical activity, body mass indexes (BMIs), regions of residence, economic levels, parents’ education levels, stress levels, smoking status, and alcohol use. A subgroup analysis of the economic groups was performed. Allergic rhinitis was positively correlated with better school performance in a dose-dependent manner (adjusted odds ratios, AOR, [95% confidence interval, CI] = 1.50 [1.43–1.56 > 1.33 [1.28–1.38] > 1.17 [1.13–1.22] > 1.09 [1.05–1.14] for grades A > B > C > D; P < 0.001). Asthma was negatively correlated with better school performance (AOR [95% CI] = 0.74 [0.66–0.83], 0.87 [0.79–0.96], 0.83 [0.75–0.91], 0.93 [0.85–1.02] for performance A, B, C, and D, respectively; P < 0.001). Atopic dermatitis was not significantly correlated with school performance. The subgroup analysis of the students’ economic levels revealed associations between allergic diseases and school performance. Compared to other allergic disorders, the asthma group had more school absences due to their symptoms (P < 0.001). School performance was positively correlated with allergic rhinitis and negatively correlated with asthma in Korean adolescents, even after adjusting for other variables. The asthma group had an increased number of school absence days, which presumably contributes to these students’ poor school performance.

## Introduction

The achievement of higher grades and better school performance is one of the most important issues in adolescence because academic achievement has a determining effect on students’ future socioeconomic levels and lifestyles. Various factors are known to be associated with school performance. Cognitive, attention, and socio-emotional factors and behaviour problems, specifically hyperactivity/impulsivity, social behaviours, and anxiety/depression, were prospectively associated with grades in math, reading, and other subjects [1,2]. In our previous study, healthy behaviours, including diet habits, were associated with school performance [3]. Higher academic performance in various subjects, including receptive vocabulary, standardized coefficients, and math skills, were related to healthy dietary habits and physical activity from childhood to adulthood [4]. Additionally, sleep disturbances and subsequent daytime fatigue may have detrimental impacts on academic performance [5].

Some diseases, such as uncontrolled asthma and allergic rhinitis with chronic, disturbing symptoms are associated with poor school performance [6–8]. Allergic disorders, including allergic rhinitis, atopic dermatitis and asthma, are prevalent and are present in up to 40% of the global population [9,10] and are present in approximately 0.2–34.1% [11] and 18–24.3% [12,13] in different ethnic groups. Although the prevalence of asthma has ceased to increase in recent years, the prevalence of allergic rhinitis is still increasing [12,14]. Industrialized lifestyles and air pollution have contributed to the increasing prevalence of allergic rhinitis in recent years [15].

Although several studies have suggested a relation between allergic diseases and school performance, most studies have focused on individual allergic diseases [8,16,17]. Moreover, many of the above described factors potentially affect school performance. Based on their common IgE-mediated pathophysiological mechanisms, allergic rhinitis, atopic dermatitis, and asthma might have some shared effects on school performance. Thus, the present study investigated the associations of common allergic disorders, including allergic rhinitis, atopic dermatitis, and asthma, with school performance among Korean adolescents. In contrast to the previous studies, we adjusted for various possible confounding factors, including physical activity, obesity, sleep time, region of residence, economic level, parental education levels, stress level, tobacco use, and alcohol use. To our knowledge, this is the first study to simultaneously delineate the associations between school performance and three allergic diseases in a large, population-based, representative study group.

## Materials and methods

### Survey

The understanding, reliability and validity of each question were investigated by the Centers for Disease Control and Prevention of Korea (KCDC) to qualify the surveys [18]. The times at which the participants fell asleep and woke up were measured in intervals of hours and 10 minutes. The duration of sleep time was calculated by subtracting the falling asleep time from the waking time. Sleep time was divided into 4 groups: < 6 hours, ≥ 6 hours, < 7 hours, ≥ 7 hours, < 8 hours, and ≥ 8 hours. The days of physical activity were defined as the number of days in the previous week on which the participant exercised for more than 60 minutes in a manner sufficient to increase the heart rate or respiration. Obesity was classified into 4 groups according to the Centers for Disease Control and Prevention Guidelines on Body Mass Index (BMI, kg/m^2^) for Children and Teens [19]: obese ≥ 95^th^ percentile; overweight ≥ 85^th^ percentile and < 95^th^ percentile; healthy weight ≥ 5^th^ percentile and < 85^th^ percentile; and underweight < 5^th^ percentile. Regions of residence were divided into 3 groups according to the administrative district: large city, small city, and rural area. The economic levels were classified into 5 levels: highest, middle high, middle, middle low, and lowest. The parents’ education levels were divided into 4 groups: college graduate or higher; high school graduate; completed middle or elementary school; unknown or no parent. The participants who did not know the education levels of their parents or who did not have a parent were not excluded to ensure that data were collected from participants with a relatively lower economic level. The self-reported stress levels of the participants were divided into 5 groups: severe, moderate, mild, a little, and no stress. The participants were asked to report the number of days they smoked in the previous month and were divided into 4 groups: 0 days per month, 1–5 days per month, 6–19 days per month, ≥ 20 days per month. The participants were asked to report the number of days they consumed alcohol, and were divided into 4 groups: 0 days per month, 1–5 days per month, and 6–30 days per month.

Participants were asked about their history of allergic rhinitis, asthma, and atopic dermatitis based on the phase three core questionnaire of the International Study of Asthma and Allergies in Childhood (ISAAC) [20]. Participants who were diagnosed by a medical doctor in the previous 12 months were recorded as positive. The participants were asked to report the number of school absences due to each disease in the previous 12 months. This variable was divided into 4 groups: no absences, 1–3 days, 4–5 days, and ≥ 7 days. The participants were asked about their academic performance in school during the previous 12 months, and were divided into 5 levels: A (highest); B (middle, high); C (middle); D (middle, low); and E (lowest).

### Study population and data collection

The Institutional Review Board (IRB) of the KCDC approved this study (2014-06EXP-02-P-A). Written informed consent was obtained from each participant prior to the survey. Because this web-based survey was performed at a school with a large population, we did not require informed consent from parents. This consent procedure was approved by the IRB of KCDC.

This cross-sectional study used the data from the Korea Youth Risk Behaviour Web-based Survey (KYRBWS). This study included one nation and used statistical methods based on designed sampling and adjusted weighted values. The KYRBWS data collected in 2009, 2010, 2011, 2012, and 2013 were analyzed. The data were collected by the KCDC. Korean adolescents in grades 7–12 voluntarily and anonymously completed the self-administered questionnaire. The validity and reliability of the KYRBWS have been documented by other studies [21,22]. The surveys evaluated data from South Korean adolescents using a stratified, two-stage (schools and classes) clustered sampling method based on the data from the Education Ministry. Sampling was weighted by statisticians, who performed the post-stratification analyses and considered the non-response rates and extreme values.

**Fig 1:**
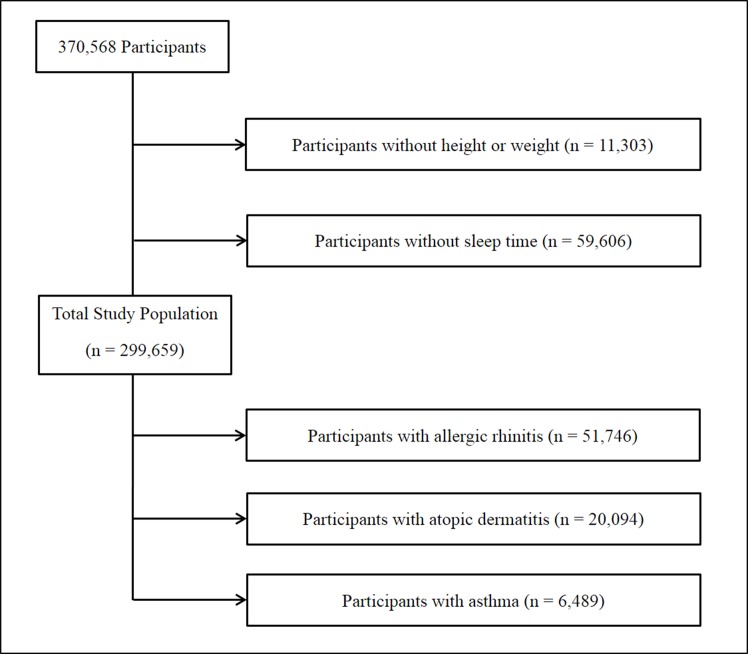
A schematic illustration of participant selection in the present study. Among a total of 370,568 participants, participants without information on height or weight (11,303), and sleep time or who sleep less than 2 hours (59,606) were excluded. The data for the 299,659 participants from whom complete data were obtained were analyzed.

Of the total of 370,568 participants, we excluded the following participants from this study: participants who did not record their height or weight (11,303 participants) and participants who did not enter their sleep time or who sleep less than 2 hours (59,606 participants). Finally, 299,659 participants (152,050 males; 147,609 females) aged 12–18 years were included in this study (Fig 1).

### Statistical analysis

The differences in the participants’ general characteristics were calculated according to their school performance levels using a linear regression analysis with complex sampling and Chi-square test with the Rao-Scott correction.

The odds ratios for allergic rhinitis, atopic dermatitis, and asthma histories with school performance were calculated using the following techniques: simple logistic regression analysis with complex sampling (unadjusted); multiple logistic regression analysis with complex sampling adjusted for age, sex, obesity, region of residence, economic level, father’s educational level, mother’s educational level, stress level, sleep time, days of physical activity, tobacco use, and alcohol use (model 1); multiple logistic regression analysis with complex sampling adjusted for the model 1 variables plus allergic rhinitis, asthma, and atopic dermatitis history (model 2).

In the subgroup analysis according to the economic level, adjusted odds ratios for allergic rhinitis, atopic dermatitis, and asthma history with school performance were calculated using a multiple logistic regression analysis with complex sampling and model 2.

School absences were compared with the Chi-square test and Rao-Scott correction.

Two-tailed analyses were conducted, and *P*-values less than 0.05 were considered significant. In addition, 95% confidence intervals (CI) were calculated. The weighted values recommended by KYRBWS were applied, and all results are presented as weighted values (S1 File). The statistical analyses were performed using SPSS ver. 21.0 (IBM, Armonk, NY, USA).

## Results

The school performance of 11.1%, 24.6%, 27.5%, 25.1%, and 11.8% of the participants was classified into the A, B, C, D, and E levels, respectively. The prevalence of allergic rhinitis, atopic dermatitis, and asthma diagnosed by a medical doctor in the previous 12 months was 18.0%, 6.7%, and 2.2%, respectively, all of which showed significant differences according to the levels of school performance (all P < 0.001) (Table 1). The participants’ mean ages, mean days of physical activity, gender, obesity status, sleep time, region of residence, family economic level, parents’ educational levels, stress level, tobacco use, alcohol consumption, and allergic rhinitis, atopic dermatitis, and asthma rates differed according to school performance (all P < 0.001) (Table 1).

**Table 1:** General Characteristics of Participants according to performance at school.

Factors			Performance at School					P-value
		Total	A	B	C	D	E	
Number								
	n	299,659	33,398	73,638	82,259	75,147	35,217	
	%	100	11.1	24.6	27.5	25.1	11.8	
Mean Age (year)		15.10	14.85	15.02	15.24	15.13	15.14	<0.001*
Physical Activity (d)		1.68	1.87	1.70	1.63	1.64	1.66	<0.001*
Sex (%)								<0.001†
	Male	50.7	56.3	50.0	49.7	49.1	52.9	
	Female	49.3	43.7	50.0	50.3	50.9	47.1	
Obesity (%)								<0.001†
	Underweight	6.7	6.6	6.3	6.7	6.9	7.6	
	Healthy	79.9	80.9	81.1	80.6	78.8	77.4	
	Overweight	10.4	9.8	10.0	10.0	11.0	11.2	
	Obese	3.0	2.7	2.7	2.8	3.3	3.8	
Sleep time (%)								<0.001†
	< 6 hours	37.7	38.3	38.2	38.8	36.1	36.8	
	≥ 6 hours, < 7 hours	25.4	26.1	25.9	25.3	25.2	24.3	
	≥ 7 hours, < 8 hours	23.0	22.6	23.4	22.5	23.5	22.5	
	≥ 8 hours	13.9	13.0	12.5	13.4	15.2	16.4	
Region (%)								<0.001†
	Large City	47.4	49.9	48.8	47.4	46.1	44.6	
	Small City	40.4	38.5	39.4	40.5	41.3	41.7	
	Rural Area	12.3	11.6	11.9	12.0	12.5	13.7	
Economic Level (%)								<0.001†
	Highest	6.1	21.4	6.4	3.3	3.0	3.8	
	Middle High	23.2	30.9	34.7	21.5	15.7	12.0	
	Middle	47.8	33.9	42.6	56.1	51.9	43.3	
	Middle Low	17.9	10.8	13.7	16.2	23.8	24.4	
	Lowest	5.1	3.1	2.6	2.9	5.6	16.5	
Education, Father (%)								<0.001†
	Unknown	16.9	9.2	11.4	15.8	21.1	29.2	
	Middle School	5.0	3.5	4.3	4.8	5.9	6.8	
	High School	35.4	26.7	33.2	37.1	38.8	36.7	
	College, or over	42.7	60.6	51.1	42.3	34.3	27.2	
Education, Mother (%)								<0.001†
	Unknown	16.6	8.8	11.0	15.5	20.8	29.3	
	Middle School	4.9	3.5	4.1	4.9	5.9	6.2	
	High School	45.7	37.9	45.0	47.8	48.1	44.3	
	College, or over	32.8	49.9	39.8	31.8	25.2	20.2	
Stress (%)								<0.001†
	Severe	11.9	10.3	9.7	10.2	12.7	19.8	
	Moderate	30.7	27.0	29.4	30.2	33.1	33.4	
	Mild	41.3	41.4	43.4	43.6	40.2	33.7	
	A little	13.8	17.5	15.3	13.8	12.1	10.6	
	No	2.3	3.7	2.1	2.1	1.8	2.5	
Smoking (%)								<0.001†
	No	89.1	94.5	93.3	91.0	85.9	75.8	
	1–5 days/month	2.8	1.5	1.8	2.5	3.7	5.1	
	6–19 days/month	1.6	0.8	1.1	1.4	2.2	2.9	
	20–30 days/month	6.5	3.1	3.2	5.2	8.2	16.1	
Alcohol (%)								<0.001†
	No	80.8	86.2	84.5	81.9	78.1	70.4	
	1–5 days/month	14.8	11.0	12.7	14.2	16.6	20.8	
	6–30 days/month	4.4	2.8	2.8	3.9	5.3	8.7	
Allergic Rhinitis (%)		18.0	22.0	19.9	17.4	16.3	15.1	<0.001*
Atopic Dermatitis (%)		6.7	6.7	6.7	6.4	6.9	7.1	<0.001*
Asthma (%)		2.2	2.1	2.2	1.9	2.2	2.7	<0.001*

*Linear regression analysis with complex sampling, Significance at P < 0.05

† Chi-square test with Rao-Scott correction, Significance at P < 0.05

The prevalence of allergic rhinitis increased as school performance improved in a dose-dependent manner (OR [95% CI] = 1.59 [1.52–1.65] > 1.39 [1.34–1.44] > 1.18 [1.14–1.23] > 1.09 [1.05–1.13] for performance A > B > C > D, P < 0.001) (Table 2). In contrast, the prevalence of asthma decreased as school performance increased (OR [95% CI] = 0.70 [0.64–0.77], 0.79 [0.71–0.88], 0.80 [0.74–0.88], 0.83 [0.76–0.90] for performance C, A, B, and D, respectively, P < 0.001). The negative relation between school performance and atopic dermatitis was also significant, but the ORs were indeterminate (OR [95% CI] = 0.94 [0.88–1.00], 0.94 [0.89–0.99], 0.90 [0.85–0.94], 0.98 [0.93–1.03] for performance A, B, C, and D, respectively). More definite relations were noted after adjusting for other variables (model 2) in both allergic rhinitis and asthma (allergic rhinitis, AOR [95% CI] = 1.50 [1.43–1.56] > 1.33 [1.28–1.38] > 1.17 [1.13–1.22] > 1.09 [1.05–1.14] for performance A > B > C > D, P < 0.001; asthma, AOR [95% CI] = 0.74 [0.66–0.83], 0.87 [0.79–0.96], 0.83 [0.75–0.91], 0.93 [0.85–1.02] for performance A, B, C, and D, respectively, P < 0.001). The prevalence of atopic dermatitis also showed negative associations with better school performance (AOR [95% CI] = 0.93 [0.87–1.00], 0.94 [0.89–0.99], 0.92 [0.87–0.97], 0.99 [0.94–1.04] for performance in A, B, C, and D, respectively, P = 0.006).

**Table 2:** Odd ratios of allergic rhinitis, atopic dermatitis, and asthma history for school performance using multinomial logistic regression analysis with complex sampling.

School Performance		Allergic Rhinitis			Atopic Dermatitis			Asthma		
		OR	95% CI	P-value	OR	95% CI	P-value	OR	95% CI	P-value
Unadjusted				<0.001*			<0.001*			<0.001*
	Performance, A	1.59	1.52–1.65		0.94	0.88–1.00		0.79	0.71–0.88	
	Performance, B	1.39	1.34–1.44		0.94	0.89–0.99		0.80	0.74–0.88	
	Performance, C	1.18	1.14–1.23		0.90	0.85–0.94		0.70	0.64–0.77	
	Performance, D	1.09	1.05–1.13		0.98	0.93–1.03		0.83	0.76–0.90	
	Performance, E	1.00			1.00			1.00		
Model 1†				<0.001*			0.049*			0.002*
	Performance, A	1.46	1.40–1.53		0.98	0.92–1.05		0.84	0.75–0.94	
	Performance, B	1.31	1.26–1.37		0.98	0.92–1.04		0.95	0.87–1.05	
	Performance, C	1.15	1.11–1.20		0.94	0.89–0.99		0.86	0.79–0.95	
	Performance, D	1.09	1.05–1.13		1.00	0.95–1.05		0.95	0.87–1.05	
	Performance, E	1.00			1.00			1.00		
Model 2‡				<0.001*			0.006*			<0.001*
	Performance, A	1.50	1.43–1.56		0.93	0.87–1.00		0.74	0.66–0.83	
	Performance, B	1.33	1.28–1.38		0.94	0.89–0.99		0.87	0.79–0.96	
	Performance, C	1.17	1.13–1.22		0.92	0.87–0.97		0.83	0.75–0.91	
	Performance, D	1.09	1.05–1.14		0.99	0.94–1.04		0.93	0.85–1.02	
	Performance, E	1.00			1.00			1.00		

* Significance at P < 0.05.

† Adjusted for age, sex, obesity, region of residence, economic level, educational level of father, educational level of mother, stress level, sleep time, day of physical activity, smoking, and alcohol consumption

‡ Adjusted for model 1 variables plus allergic rhinitis, asthma, and atopic dermatitis history

According to the subgroup analysis of the economic levels, the dose-dependent relationships between allergic rhinitis and school performance were maintained in each economic level (all P < 0.001) (Table 3). The relationships between asthma and school performance also showed improved relations (highest, AOR [95% CI] = 0.44 [0.32–0.61], 0.57 [0.41–0.79], 0.51 [0.36–0.74], 0.58 [0.38–0.87] for performance A, B, C, and D, respectively, P < 0.001; middle high, AOR [95% CI] = 0.74 [0.57–0.98], 0.83 [0.54–1.06], 0.85 [0.65–1.10], 1.05 [0.82–1.34] for performance A, B, C, and D, respectively; P = 0.009). The prevalence of atopic dermatitis was not significantly associated with school performance at each economic level.

**Table 3:** Subgroup analysis of adjusted odd ratios of allergic rhinitis, atopic dermatitis, and asthma history for school performance according to the economic level.

Economic Level		Allergic Rhinitis			Atopic Dermatitis			Asthma		
		AOR	95% CI	P-value	AOR	95% CI	P-value	AOR	95% CI	P-value
Highest				<0.001*			0.714			<0.001*
	Performance, A	1.29	1.09–1.52		0.95	0.74–1.21		0.44	0.32–0.61	
	Performance, B	1.27	1.07–1.51		0.88	0.68–1.13		0.57	0.41–0.79	
	Performance, C	1.11	0.92–1.33		0.92	0.69–1.22		0.51	0.36–0.74	
	Performance, D	1.00	0.83–1.20		1.00	0.75–1.34		0.58	0.38–0.87	
	Performance, E	1.00			1.00			1.00		
Middle, high				<0.001*			0.096			0.009*
	Performance, A	1.45	1.31–1.61		0.86	0.73–1.02		0.74	0.57–0.98	
	Performance, B	1.28	1.16–1.40		0.87	0.75–1.01		0.83	0.65–1.06	
	Performance, C	1.13	1.02–1.24		0.83	0.72–0.97		0.85	0.67–1.10	
	Performance, D	1.03	0.93–1.14		0.94	0.80–1.10		1.05	0.82–1.34	
	Performance, E	1.00			1.00			1.00		
Middle				<0.001*			0.352			0.174
	Performance, A	1.52	1.42–1.63		0.96	0.86–1.07		0.81	0.66–0.98	
	Performance, B	1.34	1.26–1.42		0.98	0.90–1.07		0.94	0.81–1.10	
	Performance, C	1.18	1.11–1.25		0.95	0.88–1.03		0.90	0.79–1.04	
	Performance, D	1.09	1.02–1.15		1.01	0.93–1.10		0.97	0.84–1.11	
	Performance, E	1.00			1.00			1.00		
Middle, low				<0.001*			0.102			0.190
	Performance, A	1.62	1.44–1.81		0.88	0.74–1.04		0.88	0.66–1.16	
	Performance, B	1.26	1.16–1.38		0.85	0.75–0.97		1.05	0.84–1.31	
	Performance, C	1.18	1.09–1.28		0.89	0.79–1.00		0.82	0.66–1.02	
	Performance, D	1.15	1.06–1.24		0.94	0.84–1.04		0.96	0.79–1.17	
	Performance, E	1.00			1.00			1.00		
Lowest				<0.001*			0.442			0.314
	Performance, A	1.44	1.18–1.76		0.89	0.65–1.20		0.88	0.57–1.36	
	Performance, B	1.52	1.30–1.80		1.16	0.93–1.44		0.89	0.63–1.26	
	Performance, C	1.20	1.03–1.40		1.08	0.89–1.33		0.68	0.48–0.97	
	Performance, D	1.11	0.98–1.25		1.03	0.88–1.21		0.87	0.68–1.12	
	Performance, E	1.00			1.00			1.00		

* Adjusted for age, sex, obesity, region of residence, economic level, educational level of father, educational level of mother, stress level, sleep time, day of physical activity, smoking, alcohol consumption, allergic rhinitis, asthma, and atopic dermatitis history

The number of school absence days increased in the asthma group compared to the allergic rhinitis and atopic dermatitis groups (P < 0.001) (Table 4). Approximately 3.2% of asthma subjects, 0.9% of allergic rhinitis subjects, and 1.0% of atopic dermatitis subjects were absent from school ≥ 7 days in the previous 12 months due to asthma, allergic rhinitis, and atopic dermatitis, respectively.

**Table 4:** School absence days in recent 12 months due to allergic rhinitis, atopic dermatitis, and asthma.

Absence	Allergic rhinitis		Atopic dermatitis		Asthma	
	N	% (95% CI)†	N	% (95% CI)†	N	% (95% CI)†
Total	51,746	100	20,094	100	6,489	100
0 day	47,374	91.7 (91.4–91.9)	18,823	94.0 (93.7–94.4)	5,117	80.3 (79.2–81.3)
1–3 days	3,437	6.6 (6.4–6.9)	900	4.2 (3.9–4.6)	898	13.6 (12.7–14.5)
4–6 days	451	0.8 (0.8–0.9)	158	0.7 (0.6–0.9)	187	3.0 (2.5–3.5)
≥ 7 days	484	0.9 (0.8–1.0)	213	1.0 (0.8–1.2)	228	3.2 (2.7–3.7)
P-values	< 0.001*		< 0.001*			

CI: confidence interval

*Chi-square test with Rao-Scott correction when comparing with asthma, Significance at P < 0.05

† Estimate prevalence

## Discussion

Allergic rhinitis was significantly correlated with better school performance in the present study. In contrast, asthma was negatively associated with better school performance. The relation between school performance and allergic rhinitis was dose-dependent. Specifically, after adjusting for possible confounders, including other allergic diseases, the power of the correlations was superior to that of the unadjusted models. These results might be partially explained by the inverse correlations between allergic rhinitis and asthma with school performance. For instance, in subjects with allergic rhinitis and asthma, the negative correlation between asthma and school performance could counteract the positive association between allergic rhinitis and school performance, and vice versa. These situations might result in the reduced correlations observed in the unadjusted models. Although various socio-economic factors, including economic levels, were adjusted as confounders, we conducted a subgroup analysis on the economic levels, because we believe the students’ economic levels could be a strong confounder of the relation between allergic diseases and school performance. The subgroup analysis based on the economic levels also revealed significant relations between allergic rhinitis and asthma with school performance.

The negative correlation between school performance and asthma could be explained by the observation that asthma subjects had the highest number of school absence days compared to participants with allergic rhinitis and atopic dermatitis. Consistent with the present results, previous studies reported that asthma, particularly severe or poorly controlled asthma, was significantly associated with a greater number of school or work absences [7,23,24]. Uncontrolled nocturnal asthma symptoms may contribute to daytime working productivity. Moreover, asthma may induce depression and emotional symptoms, thereby causing a detrimental effect on school performance [16]. Asthma subjects were also more susceptible to depression and emotional symptoms in previous studies [16,25]. Atopic-related responses, which could exist concomitantly with other allergic disorders, such as allergic rhinitis and atopic dermatitis, as well as non-atopic-mediated inflammatory reactions or stress responses, which might be more profound in asthma subjects compared with subjects with other allergic disorders, due to disease severity. These responses are presumed to impact depression and, in turn, the poor school performance in this study [16,25]. An inverse causal relation between depression and allergic diseases is also probable. The mediators of atopy may be up-regulated in response to depressive emotional situations [26,27]. Furthermore, poorly controlled asthma has detrimental effects on adolescents’ and their caregivers’ quality of life, resulting in limitations in performance and emotional functioning and subsequent poor educational support [7].

On the other hand, school performance was positively associated with allergic rhinitis in a dose-dependent manner in the present study. In contrast to our results, allergic rhinitis was suggested to be related to poor school performance in previous studies [8,17]. Bothersome symptoms of allergic rhinitis, including rhinorrhoea, postnasal drip, sneezing, and nasal obstruction disturb, sleep hygiene and induce daytime fatigue, which may hinder students’ abilities to concentrate on school work and impair cognitive function [28–32]. Comorbidities of allergic rhinitis, such as sinusitis and secondary Eustachian tube dysfunction, may result in hearing impairments and could be a handicap while students are attending classes [17]. Emotional distress, depression, and disturbances in social interactions due to allergic symptoms may hamper school performance [33]. Moreover, adverse effects of allergic medications, such as the central and anticholinergic effects of first generation antihistamines, may distract patients in the classroom [8]. However, these detrimental effects of allergic rhinitis on school performance may be noted in subjects with severe or uncontrolled allergic rhinitis. The present study comprised a wide range of severities and types of allergic rhinitis. These adverse effects of allergic rhinitis on school performance may be minimal in mild or well-controlled cases. Additionally, most previous studies on the negative association between allergic symptoms and academic performance did not consider various confounders, which are compared with a large number of variables in the present study.

In accordance with our results, a recent cross-sectional study of 31,802 high school students also reported a positive association between better school performance and allergic rhinitis (1.20) but reported a negative correlation between school performance and asthma (0.89) [34]. This positive relation between allergic rhinitis and better school performance may be attributable to the high socio-economic status of allergic rhinitis patients. Allergic rhinitis is more common in developed countries and urban areas [35]. The hygiene hypothesis, which suggests a high risk of allergic diseases in only children and firstborns, has been supported by epidemiological studies [34]. Although we considered socio-economic factors by adjusting for economic levels and parental educational levels, we could not exclude the hidden socio-economic variables related to allergic rhinitis, such as the subjects’ living environments and dietary habits, which are associated with better school performance. We conducted a subgroup analysis based on the economic levels to minimize the influence of these economic factors, and a clear dose-dependent relation between allergic rhinitis and better school performance was still observed.

The psycho-emotional characteristics of the adolescents with allergic rhinitis may be related to the improved school performance in this group [36]. As reported in previous studies allergic rhinitis may have adverse emotional effects, including anxiety, interpersonal sensitivity, hostility, impulsiveness, depression, and a low threshold for stress [36]. These detrimental emotional traits may have negative effects on school performance. However, it was reported that patients with allergic rhinitis tended to have a type C personality, which includes more anxious, dependent and anankastic characteristics [37]. These compliant and perfectionistic traits may allow patients with allergic rhinitis to perform better in school. Conversely, better school performance was associated with more stress or emotional labour from school work, which is suggested to be associated with allergic rhinitis. Emotional labour or stress, in which emotions are concealed at work, was suggested to be related to allergic rhinitis [38]. Stress was correlated with allergic rhinitis and other allergy-associated symptoms in adolescents [39]. Although a causal relationship could not be determined, a plausible mechanism through the hypothalamus-pituitary-adrenal axis was hypothesized to explain the contributions of stress to allergic disorders [40]. Augmented secretion of corticoids in the adrenal cortex in response to stress might deregulate cytokine profiles, which favour interleukin (IL)-4, IL-10, and IL-13, but diminish IL-12. These changes in cytokine release stimulate Th2 cells and inhibit Th1 cells, thereby resulting in allergic reactions [40]. Furthermore, prolonged exposure to stress was reported to aggravate inflammatory responses via cross-talk between the nervous and immune systems through neurotransmitters on mast cells [41]. Annoying symptoms of allergic diseases and school work may be considerable stressors. In turn, these stressors provoke allergic reactions. Therefore, the relations between allergic rhinitis and stress, including school work stress, can lead to vicious cycles.

Atopic dermatitis showed a significant but weak negative correlation with school performance in our study. No previous study has evaluated the effect of atopic dermatitis on school performance. Eczema was suggested to be related with worse sleep quality and neurocognitive deficits, such as verbal comprehension, perceptual reasoning and working memory, in children [42]. However, although approximately 43–45% of subjects with atopic dermatitis developed asthma and/or allergic rhinitis, approximately 87.2% of subjects with atopic dermatitis showed resolved symptoms in a prospective study [43]. This good prognosis of atopic dermatitis and the adjustment for asthma and allergic rhinitis in the present study might mitigate the correlation between atopic dermatitis and school performance.

The present study used a cross-sectional design, which limits the interpretation of our results with respect to the causalities between allergic disorders and school performance. Because the presence of allergic disorders and the levels of school performance were surveyed using self-reported questionnaires, recall bias may be inevitable. We could not classify the severity and types of allergic disorders. Although we considered numerous covariates of socio-economic, demographic, physical, and stress factors, we could not exclude the possibility that the other confounding variables could influence the relations between allergic disorders and school performance. Nonetheless, the present study has some strengths. We examined 299,659 participants who represent a nationwide adolescent population in Korea. The qualified surveys, which included validated items, strengthened the reliability of our results [20]. Moreover, this study adjusted for age, physical activity, sex, obesity, sleep time, region of residence, economic level, parental education levels, stress levels, smoking, alcohol use, and other allergic disorders. To the best of our knowledge, this list might encompass the largest number of variables used to investigate the factors related to allergic disorders. Foremost, this study is the first to explore the relationship between allergic disorders and school performance by concurrently considering allergic rhinitis, asthma, and atopic dermatitis. The exact causal relations and the pathophysiologic mechanisms of the associations should be determined in future studies.

## Conclusion

Allergic rhinitis was positively associated with better school performance in adolescents. In contrast, asthma showed negative correlations with better school performance in these groups. The relationships of allergic rhinitis with school performance were dose-dependent and persisted even after adjusting for age, physical activity, sex, obesity, sleep time, region of residence, economic level, parental education levels, stress level, smoking, alcohol use, and other allergic disorders. Increased numbers of school absence may contribute to the negative correlation between asthma and school performance. Therefore, school life should be a particular concern for the asthmatic adolescent.

## Supporting information

S1 File The analytic methods of weighting. (DOCX)
