# Supplementary material for: Allergic rhinitis, atopic dermatitis, and asthma are associated with differences in school performance among Korean adolescents
Source: PLoS One. 2017 Feb 16;12(2):e0171394. doi: 10.1371/journal.pone.0171394 (PMC5312966; doi:10.1371/journal.pone.0171394)
Supplement: S1 File — (DOCX) [file pone.0171394.s001.docx]

**S1 File The analytic methods of weighting**

In this study, the participants were weighted using this method to represent mother populations. Weighted values were calculated using extraction rate, response rate, and correction rate.

Weight = (1/extraction rate) * (1/response rate) * correction rate

1/extraction rate = (1/extraction rate of school) * (1/extraction rate of class) = (the number school in mother population/the number of school in sample) * (the number of class in each grade)

1/response rate = The number of total participants in each grade/the number of responded participants in each grade

Correction rate = The number of student in each sex, school, and grade in mother population/ The number of student in each sex, school, and grade in survey region
